# Monitoring subsurface soil displacement in karst collapse by designing a MEMS-based spherical monitoring device

**DOI:** 10.1038/s41598-025-00821-w

**Published:** 2025-05-12

**Authors:** Di Wu, Taiming Liang, Fan Jiang, Yanxin Yang, Qingpeng Pei, Jianjian Wu

**Affiliations:** 1 School of Architecture and Transportation Engineering, Guilin University of Electronic Technology, Guilin, 541004 China; 2 Natural Resources Ecological Restoration Center of Guangxi Zhuang Autonomous Region, Nanning, 530028 Guangxi China; 3 School of Civil Engineering, Sichuan University of Science & Engineering, Zigong, 643002 China; 4 Guangxi Guiguan Electric Power Co., Ltd., Nanning, 530029 China

**Keywords:** Natural hazards, Engineering

## Abstract

To address challenges in monitoring subsurface soil displacement in karst collapse areas, a MEMS-based spherical monitoring device was developed to accommodate complex subsurface monitoring environments. Six sets of fixed-distance tests were designed to verify the flexibility of the spherical monitoring devices in moving with the sub-surface soil. Furthermore, four indoor model tests were conducted to acquire displacement data, which were developed from MEMS sensors and MEMS sensors with spherical shell arranged at identical positions. PIV was employed to analyze the monitoring accuracy of the MEMS spherical monitoring device and MEMS sensor in karst collapse model tests, to further evaluate the practicality of the spherical monitoring devices for monitoring in karst collapse. The results of the fixed-distance tests indicate that the spherical monitoring device effectively mitigates the influence of soil pressure on the monitoring cables. Model test results show that, in comparison to MEMS sensor, the MEMS spherical monitoring device exhibits a reduced average relative error of 23.09% in stable zone displacement measurements and 18.87% in the subsidence zone. This suggests that the MEMS-based spherical monitoring device better captures variations in sub-surface soil displacement. This paper provides a new insight for karst collapse monitoring and the application of MEMS sensors in geotechnical engineering.

## Introduction

Karst collapse refers to the deformation and failure of the rock and soil mass above karst caves, caused by natural or human factors, resulting in the formation of surface collapses^1,2^. The phenomenon significantly hampers the economic and social development in karst regions. However, the covert nature of karst development and the suddenness of collapse make conventional methods of ground deformation monitoring challenging to achieve monitoring objectives^3,4^. Specific monitoring tools are used to monitor the deformation of subsurface soil. Currently, traditional methods for monitoring karst collapses primarily include optical fiber sensing technology, geological radar monitoring, and time-domain reflectometry coaxial cable monitoring^5^. The high cost and difficulty in installation and protection make optical fiber sensing monitoring equipment slow to be widely adopted in engineering applications^6^. Geological radar monitoring lacks real-time capabilities and is susceptible to interference from surrounding electromagnetic waves^7^. Additionally, it is known to have limitations in monitoring sinkholes at depths exceeding 15 m^8^. Time-domain reflectometry coaxial cable monitoring technology has the advantages of low cost and good interference resistance, but the equipment can only monitor characteristic signals when subjected to shear or tensile forces, resulting in low monitoring effectiveness during the collapse development process^9^.

In recent years, as the manufacturing processes of MEMS sensors continually advance, more precise MEMS inertial sensors have been developed. These sensors have advantages such as small size, low cost, low power consumption, and easy installation, and they are capable of outputting acceleration, tilt, and other posture data of the measured object without relying on any external information. Due to these attributes, MEMS inertial sensors have gained increased attention from researchers in various fields, including underwater measurements, military applications, and aerospace.

In the field of subsurface soil deformation monitoring of geotechnical engineering, numerous researchers have designed various monitoring devices and methodologies based on MEMS inertial sensors, taking into account different engineering scenarios. Tao^10^ developed a collaborative measurement system, utilizing a dual MEMS sensor structure. This system transforms spatial deflection angles and torsional angles acquired from the sensors into endpoint coordinates of the sensor array, employing these coordinates to compute the deformation and settlement of the soil inside the dam. Zhu^11^ used MEMS sensors to monitor the inclination angle of an object to monitor the deformation of landslides, and combined MEMS sensors with other types of sensors to explore the deformation mechanism of landslides. Wu^12^ used MEMS sensors in a model test to obtain soil deformation data of soil bodies at different depths of the foundation pit. Moreover, Jiao^13^, Freddi^14^, and Shentu^15^ each designed flexible soil deformation monitoring devices with multiple measurement units based on MEMS inertial sensors. In summary, most scholars focus on refining monitoring devices based on MEMS inertial sensors to measure the internal displacement of soil through tilt angle analysis. However, studies investigating subsurface displacement from an inertial measurement perspective remain limited. In practical engineering applications, displacement data obtained by sensors are often prone to inaccuracies due to environmental disturbances during inertial measurements^16–27^, leading to unreliable monitoring results. Therefore, it is imperative to investigate karst collapse displacement from the perspective of inertial measurement and develop a novel monitoring methodology characterized by low cost and operational convenience.

This study proposes a MEMS-based spherical monitoring device. A comparative fixed-distance test was conducted using the MEMS spherical monitoring device and MEMS sensors. Building upon the principles of the trapdoor test, karst collapse model experiments were implemented to simulate subsurface deformation dynamics. In these experiments, particle image velocimetry (PIV) cameras were employed to analyze the monitoring accuracy of the MEMS spherical device and MEMS sensors, enabling a quantitative comparison of their performance under controlled collapse conditions. The experimental findings demonstrate that the MEMS spherical monitoring device exhibits superior monitoring performance compared to conventional MEMS sensors. Through acceleration-to-displacement conversion, this approach provides a novel methodology for karst collapse monitoring applications, enabling long-term observation while maintaining cost-effectiveness.

## Design of the device and calibration test

### The design of the new monitoring device

Inertial sensors are devices that measure physical quantities based on the principles of first law of Newton. They rely on the effects of inertia generated during the motion or transient motion state of an object to detect information such as acceleration, angular velocity, and direction. Therefore, it is imperative for inertial sensors to ensure certain synchronicity with the object’s motion state to effectively acquire data^28^.

With the continuous development of MEMS packaging technology, MEMS inertial sensors have gradually become mainstream, MEMS inertial sensors are mainly made of accelerometer, gyroscopes and other sensors collection, the principle is to use accelerometer and gyroscopes to sense the acceleration and angular velocity of the object movement, through the acquisition of acceleration and angular velocity integration, and then obtain speed, angle, displacement and other information. To better capture the real deformation information of underground soil, the research team developed a MEMS-based spherical monitoring device.

**Fig. 1:**
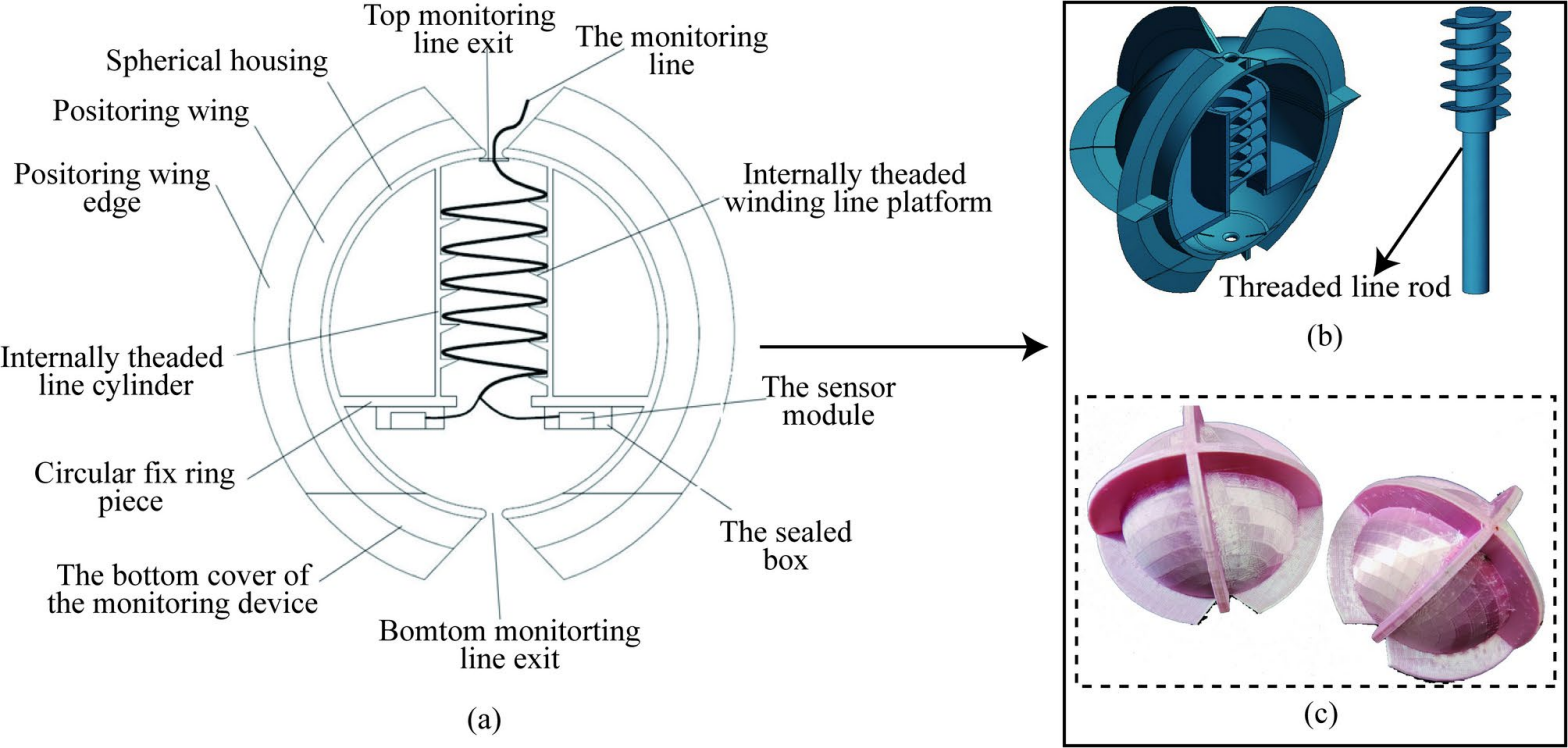
The structural design of the spherical monitoring box. (a) Two-dimensional structural design drawing of the spherical monitoring box; (b) three-dimensional structural design drawing of the spherical monitoring box; (c) the structural design physical drawing of the spherical monitoring box.

**Fig. 2:**
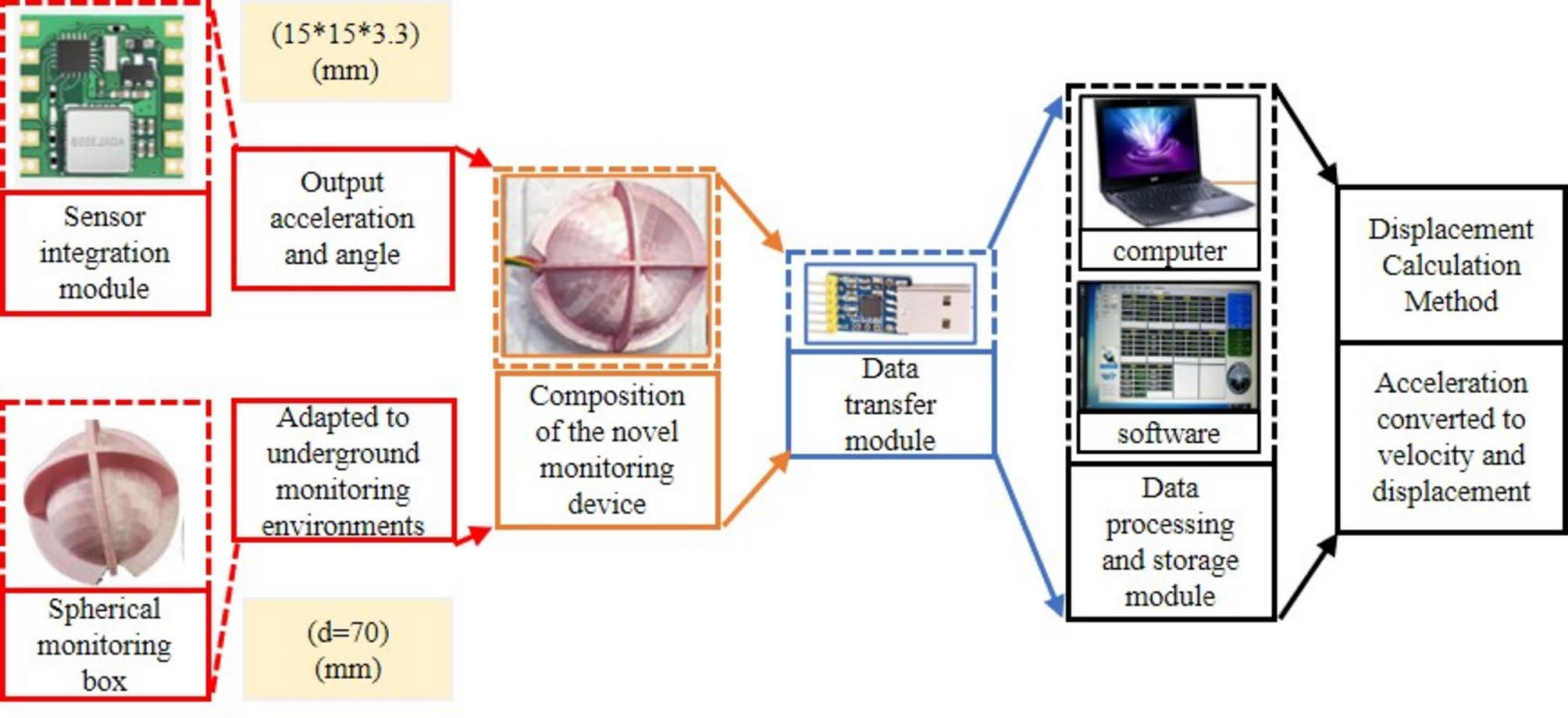
Composition and working principle of the spherical monitoring device.

The encapsulated housing of the spherical monitoring device (spherical monitoring box) has a semi-open spherical design with a spherical diameter of 70 mm. Three orthogonal positioning wings are on its outer surface. The edges of these wings enhance the ability to embed in the soil, improving the synchronization of the monitoring device with soil movements. Internally, the monitoring device comprises an internally threaded line cylinder, a circular fixed ring piece, a threaded line rod, and an internally threaded line platform. The circular fixed ring piece secures the internally threaded line cylinder and inertial measurement module. The threaded line rods and the internally threaded line platform, situated within the cylinder, twine and place the monitoring lines inside the monitoring device. This structure prevents the direct compression of the monitoring lines by the soil, thereby avoiding any interference with the movement of the monitoring device., as shown in Fig. 1. The MEMS module HWT901B is an inertial measurement module for spherical monitoring devices that simultaneously measures three-axis acceleration, three-axis angular velocity, and three-axis angle of the moving soil, and it is available in a cube with dimensions of 15*15*3.3 mm. The measurement module and spherical monitoring box form the soil deformation sensing module. The sensed soil deformation data is uploaded to the computer through the data transmission module, The data reception and storage are carried out on the upper computer software. The acceleration of soil movement can be converted to soil displacement by the method of displacement calculation at a later stage. The composition and working principle of the spherical monitoring device is shown in Fig. 2.

### Displacement calculation methods

**Fig. 3:**
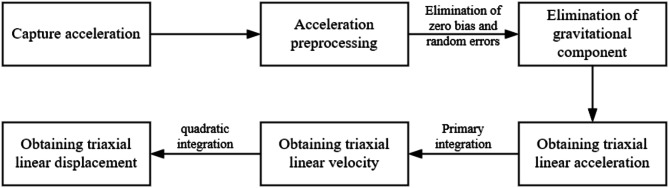
The flowchart of the displacement calculation method.

MEMS sensors directly output acceleration signals, necessitating preprocessing of the raw acceleration data. During preprocessing, the mean removal method is employed to eliminate zero-bias errors in the acceleration signal^29^. Subsequently, a wavelet threshold denoising function is introduced to remove random noise components from the acceleration signal^30^. The Kalman fusion algorithm is utilized to obtain precise real-time attitude angles of the measured object^31^. The gravitational components are then eliminated from the acceleration data using the direction cosine matrix method^32^, yielding linear acceleration. Displacement data are derived through the second integration of linear acceleration^33^. To address the accumulation of error terms inherent in numerical integration, this study adopts a Lagrange interpolation polynomial ranging algorithm with four interpolation nodes^34–36^. The flowchart of the displacement calculation method is illustrated in Fig. 3.

### Fixed-distance test

**Fig. 4:**
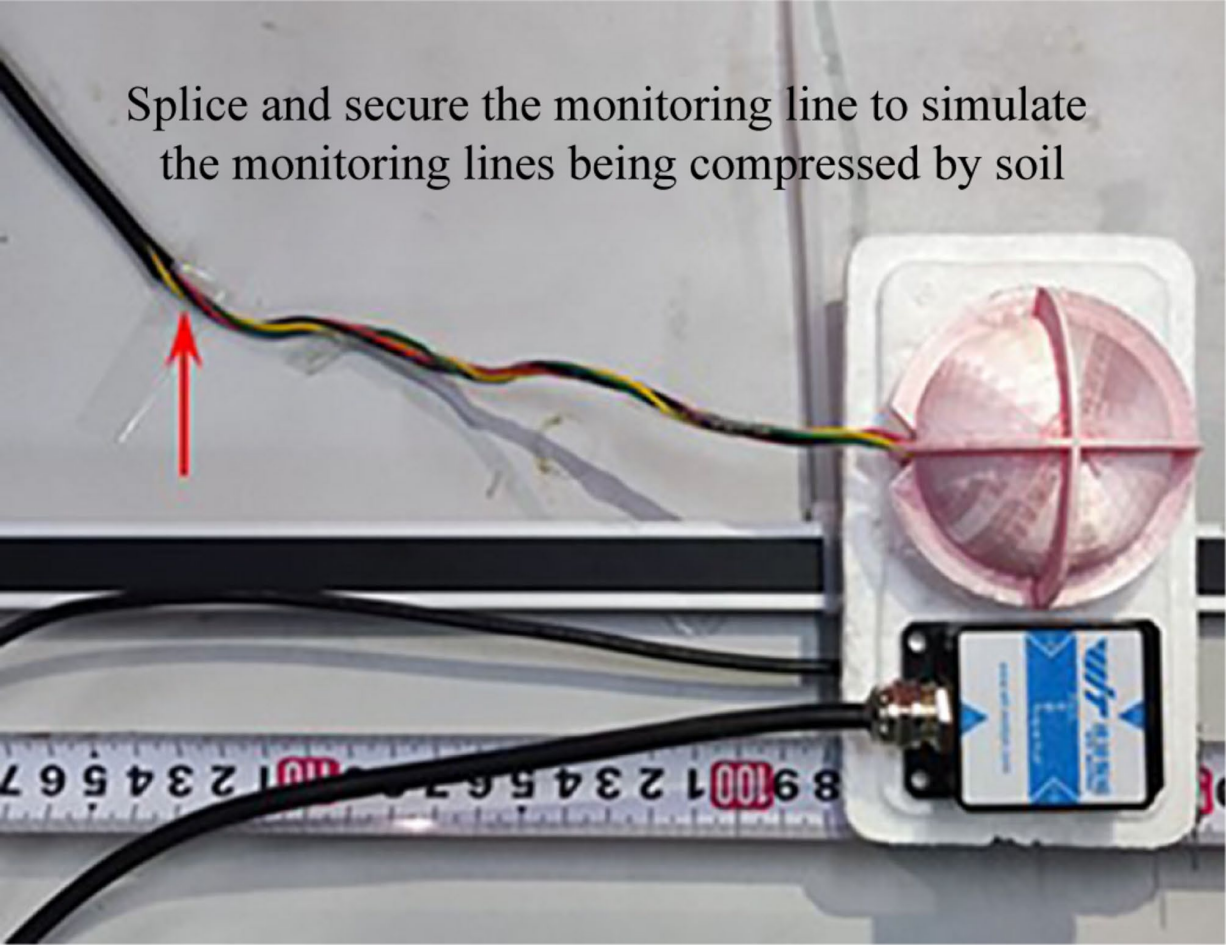
The figure of the fixed-distance test.

To investigate the applicability of the algorithm and the flexibility of the movement of the spherical monitoring device in the subsurface soil, six fixed-distance tests were designed. The tests fixed the MEMS sensor and MEMS spherical monitoring device on the same rigid plate. The MEMS sensor, representing the ideal condition, was unconstrained by external forces and could move freely with the rigid plate, simulating the ideal state when the sensor was embedded in the soil without the signal line being compressed by the soil. The external surface of the spherical monitoring device was pasted and fixed with the monitoring line, simulating the restraining effect of soil pressure and friction on the external wire of the device. Tension was applied to the rigid plate, causing the MEMS sensor and MEMS spherical monitoring device to move together. During the pulling process, a camera recorded real-time video of the displacement of the sensors. The position of the tape measure where the sensor was located in the video served as the reference for actual displacement, as shown in Fig. 4.

**Fig. 5:**
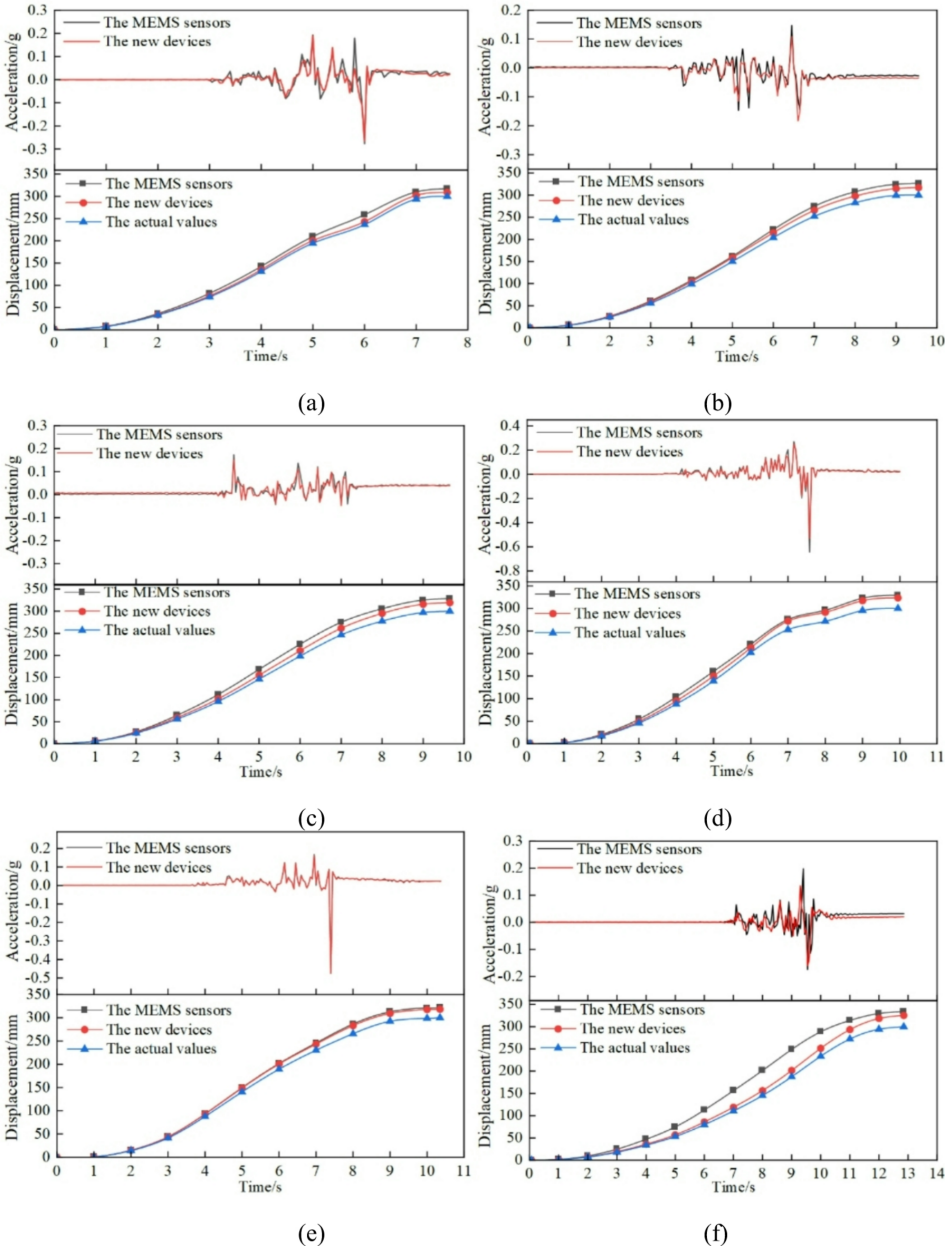
The dates of Acceleration/displacement in six groups of fixed-distance tests. (a) Acceleration/displacement data for test a; (b) Acceleration/displacement data for test b; (c) acceleration/displacement data for test c; (d) acceleration/displacement data for test d; (e) Acceleration/displacement data for test e; (f) acceleration/displacement data for test f.

For six groups of fixed-distance tests, the acceleration curve and displacement curve obtained from the MEMS sensor and the MEMS spherical monitoring device are relatively close, indicating that the design of the spherical monitoring device can make the sensors move flexibly when the upper layer of the cable under pressure. Taking the final displacement values of the MEMS sensor and MEMS spherical monitoring device for relative error analysis, it can be seen that in the six groups of tests, the average value of the relative error of the MEMS spherical monitoring device is 6.73 in percentage, the average value of the relative error of the MEMS sensors is 8.76 in percentage, and they are close to the actual value of 300 mm, which indicates that the integration algorithm is suitable for calculating the displacement of the MEMS sensors. The results of six groups of fixed-distance tests are shown in Fig. 5, and the relative error value of the final displacement value can be referred to in Table 1.

**Table 1:** The relative error value of final displacement values of the sensors in the fixed-distance tests.

Test	Time/s	Integral displacement		Relative error		Mean relative error	
		MEMS Sensor /mm	MEMS spherical monitoring device /mm	MEMS Sensor /%	MEMS spherical monitoring device /%	MEMS sensor /%	MEMS spherical monitoring device /%
a	7.60	317.3	308.8	5.77	2.93	8.76	6.73
b	9.55	326.9	317.1	8.97	5.70		
c	9.65	328.5	319.1	9.50	6.37		
d	9.95	329.3	323.4	9.77	7.80		
e	10.35	321.8	318.3	7.27	6.10		
f	12.85	334.1	325.5	11.36	8.50		

### Verification mode test

#### The design of the model test

To further validate the practicality of the designed soil displacement measurement monitoring device in karst col-lapse areas, model experiments were conducted for verification. The experiment focused on the road collapse on the West Ring Road in Hechi City^37^ in Guangxi, China. Indoor model tests were designed, with the actual collapse replicated in a scaled-down model box at a ratio of 1:5.

The model box is constructed by welding elongated steel plates together. The box has a total length of 1.5 m, a width of 0.6 m, and a height of 1.5 m. The front face of the box is made of 25 mm thick glass, allowing for easy observation of soil movement during filling. The bottom of the box serves as the soil collapse device, with a width of 0.3 m corresponding to the subsidence zone. Stable platforms on both sides of the Subsidence zone, each measuring 0.6 m, correspond to the stable zone. The interior of the device employs a sliding screw mechanism for vertical movement, with anti-slip grooves inside the screw, enabling precise control of collapse displacement. The maximum settlement of the entire device is 60.0 mm. Through this collapse device, the soil inside the box will collapse as the settlement plate moves downward, simulating the collapse caused by various collapse-inducing forces in natural karst terrain.

#### The deployment of sensors

**Fig. 6:**
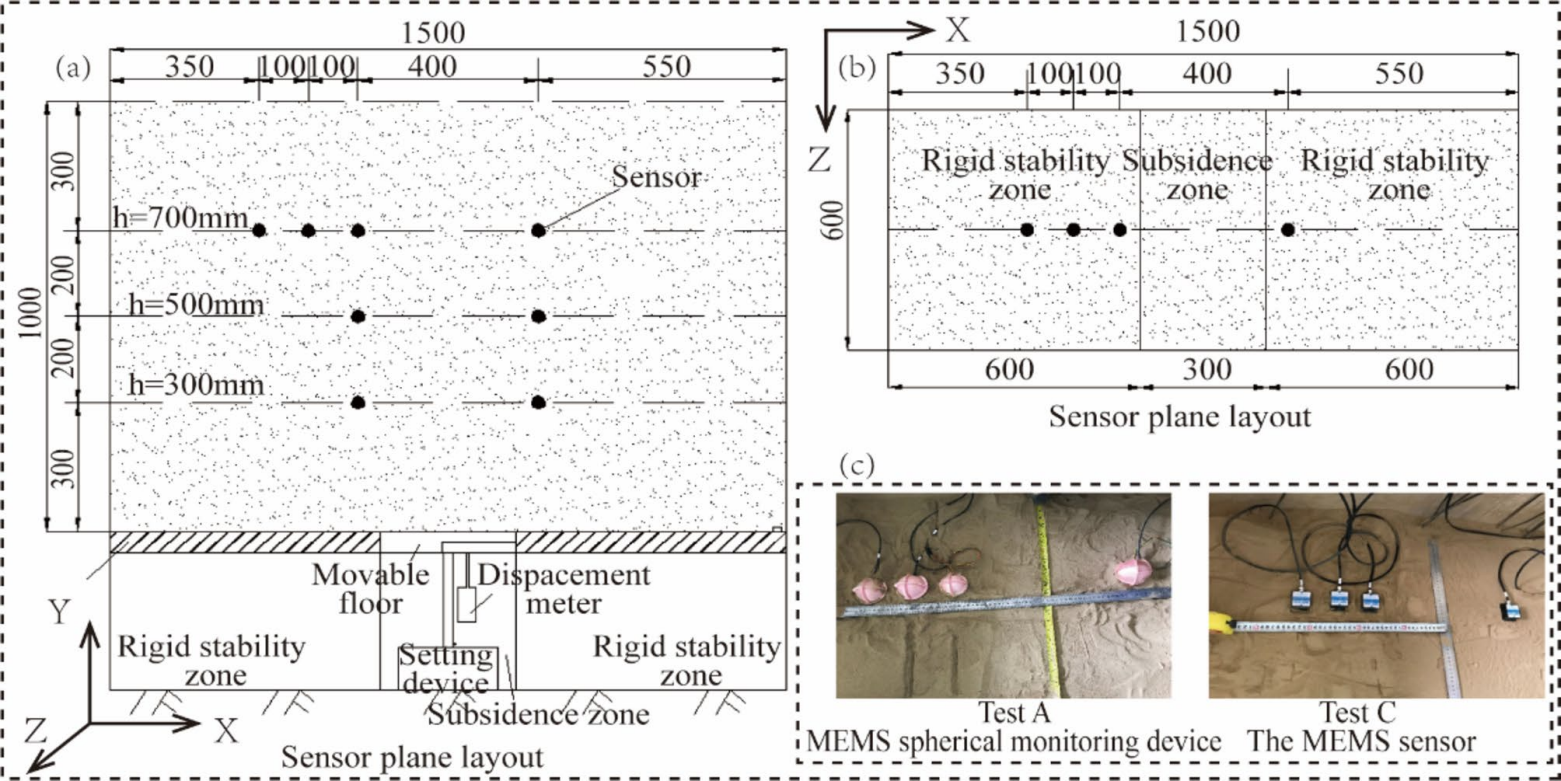
Layout plan of sensors in the Stable zone (a) Sensors facade layout; (b) Sensors plane layout (c) Realistic arrangement of sensors.

Depending on the monitoring area and sensor type, four groups of model tests were conducted for collapse monitoring. In test A and test C, MEMS sensors were installed across three layers, all within the stable zones. In test B and test D, MEMS sensors were also spread across three layers; but primarily located within the Subsidence zones. Tests A and B consisted of MEMS spherical monitoring device, while tests C and D made use of MEMS sensors. The specific sensor distribution is illustrated in Fig. 6 (for Tests A and C) and 7 (for Tests B and D).

**Fig. 7:**
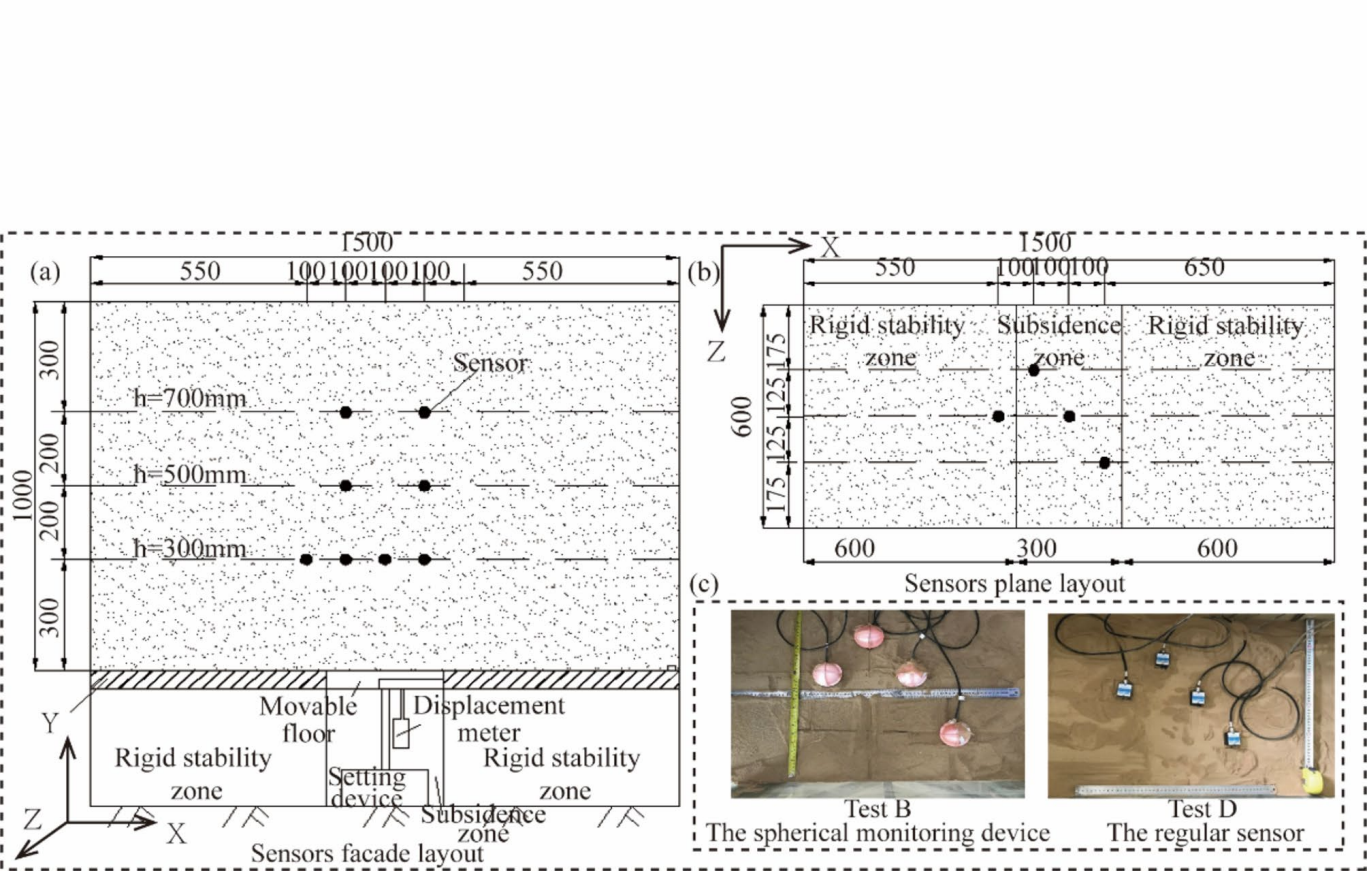
Layout plan of sensors in the Subsidence zone. (a) Sensors facade layout; (b) sensors plane layout; (c) realistic arrangement of sensors.

### The specific steps of the model test

The specific steps are as follows:

1. Before the test, polytetrafluoroethylene (PTFE) film is placed inside the model box (apart from the glass surface) to reduce friction between the sand and the box and ensure the sand complies with the boundary constraints. The sensors are calibrated to measure accelerations, angles, and the magnetic field.
2. The placement of MEMS sensors is carried out, with the X-axis parallel to the glass surface of the model box, and the right side is considered the front. The Y-axis is perpendicular to the glass surface of the model box, as shown in Fig. 6c and Fig. 7c. After the placement, further calibration is conducted to zero the sensor angles and accelerations. The X and Y-axis accelerations are set to 0, while the Z-axis acceleration is set to 1.
3. The fill soil was slowly sprinkled into the model test box, with each layer compacted to a thickness of 100 mm. The compaction coefficient was set at 0.98. The first layer of sensors was placed when the fill reached a depth of 300 mm. The soil was left undisturbed for one hour after reaching a height of 1.000 m.
4. The displacement sensor was installed beneath the movable bottom plate to monitor the settlement of the bottom plate during the test in real-time. A mechanical-electrical dial gauge with a sensitivity of 0.01 mm and a maximum range of 55 mm was selected as the displacement sensor.
5. The particle image velocimetry (PIV) device was installed and calibrated. Once all the equipment started recording data, the lead screw was rotated to initiate a downward movement of the settlement baseplate at 15 mm/min speed until the dial gauge reached 50 mm. Subsequently, the recording of data from the MEMS sensors, PIV, and dial gauge was stopped.
6. After the completion of the test, the sand was excavated, and the buried MEMS sensors were extracted. The movable baseplate at the bottom was restored to its initial position, and the displacement gauge was placed and connected to the static strain gauge. The above steps were repeated until all the tests were completed.

**Fig. 8:**
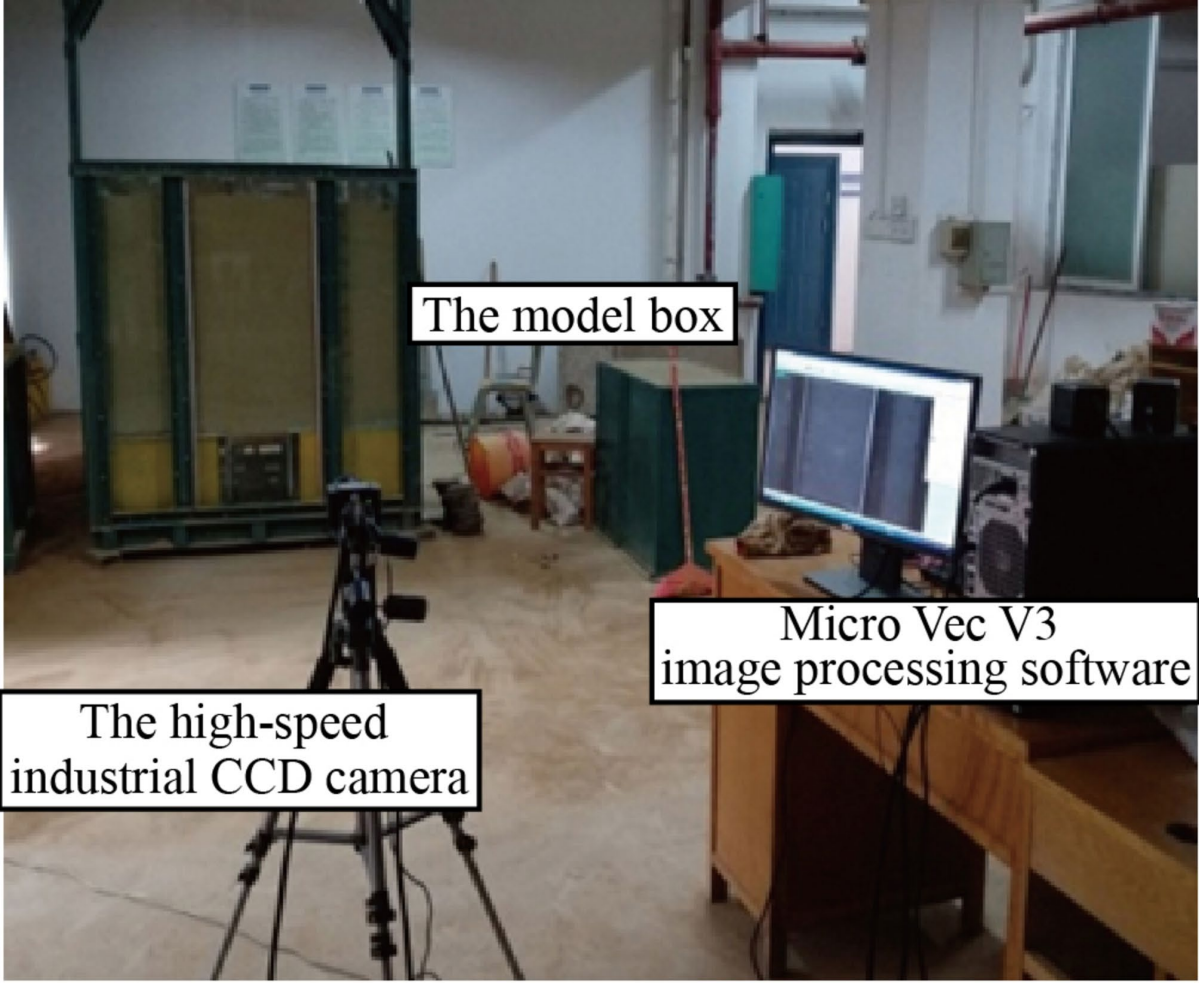
The figure of the model test.

The setup of the model test is shown in Fig. 8.

### Test result and analysis

Considering the positions and types of sensors, the time-domain integration algorithm was used to process the acceleration and tilt data obtained from indoor-scale model tests. The integrated results were compared to analyze dis-placements and determine differences in the applicability of the MEMS spherical monitoring device compared to MEMS sensors in karst collapse monitoring. Additionally, the collapse patterns of karst collapse were summarized. Finally, by comparing with cloud images captured by the PIV system, the practicality of the MEMS spherical monitoring device for measuring subsurface soil displacement in karst Subsidence zones was further evaluated.

### Displacement analysis of stable zone

The sensors were installed in three layers at layout heights of h = 300, 500, and 700 mm. Overall, the vertical soil displacements in the stable zone remained within 8 mm, while the lateral displacements did not exceed 15 mm. These results indicate a more significant lateral than vertical impact of the collapse on the subterranean soil in the stable zones. the sensors labeled A7, A8, C7, and C8, located at the horizontal widths of L = 350 mm and 450 mm, showed negligible displacements in both vertical and lateral directions, thus, their data are not displayed in the figures.

Based on the collapse model test setup, the vertical displacement is considered as a Planar problem. Data changes are observed only in the X and Z axes of the MEMS sensors, and the Y-axis displacement is not taken into account. Therefore, it can be treated as a two-dimensional motion in space. Additionally, since the model test simulates the top-down collapse of the soil in the karst collapse process, the positive direction is oriented downwards, and the vertical axis is plotted in a downward direction on the figures.

**Fig. 9:**
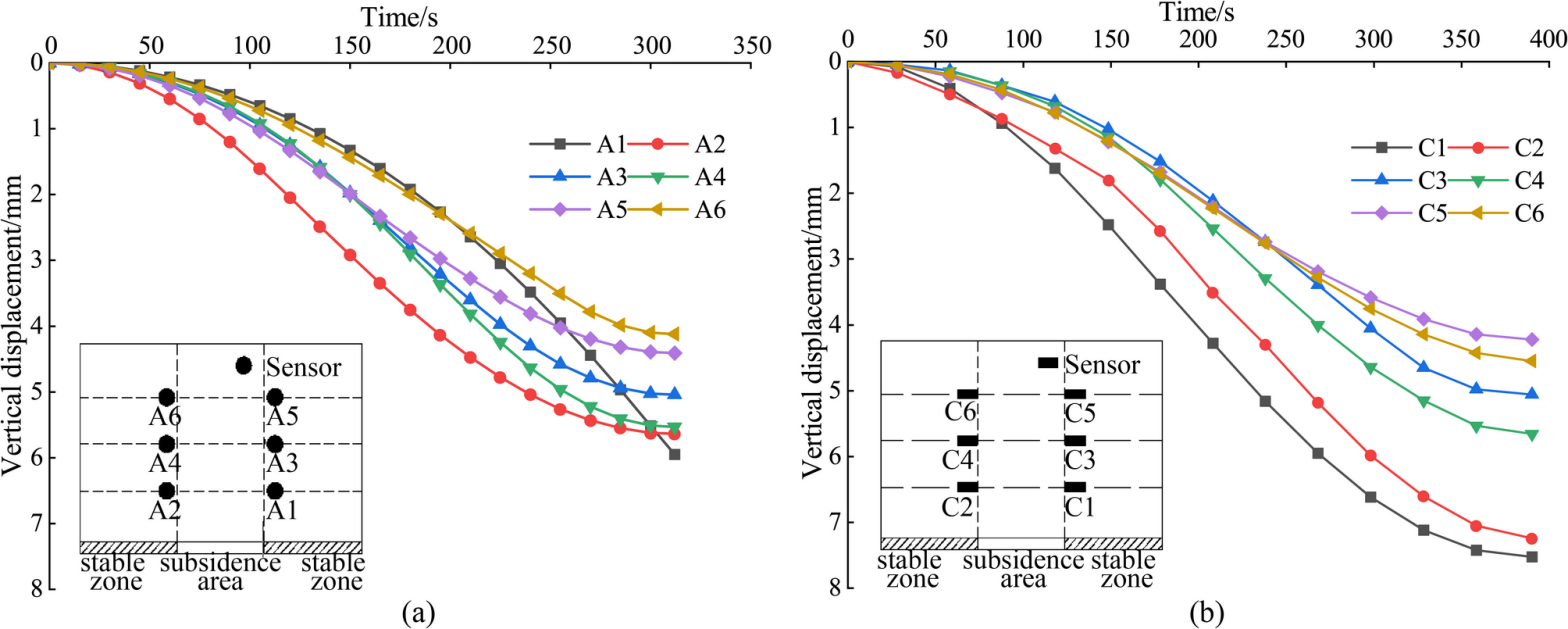
Analysis of vertical displacement in the stable zone. (a) The vertical displacement of the MEMS spherical monitoring devices in the stable zone; (b) the vertical displacement of the MEMS sensors in the stable zone.

**Fig. 10:**
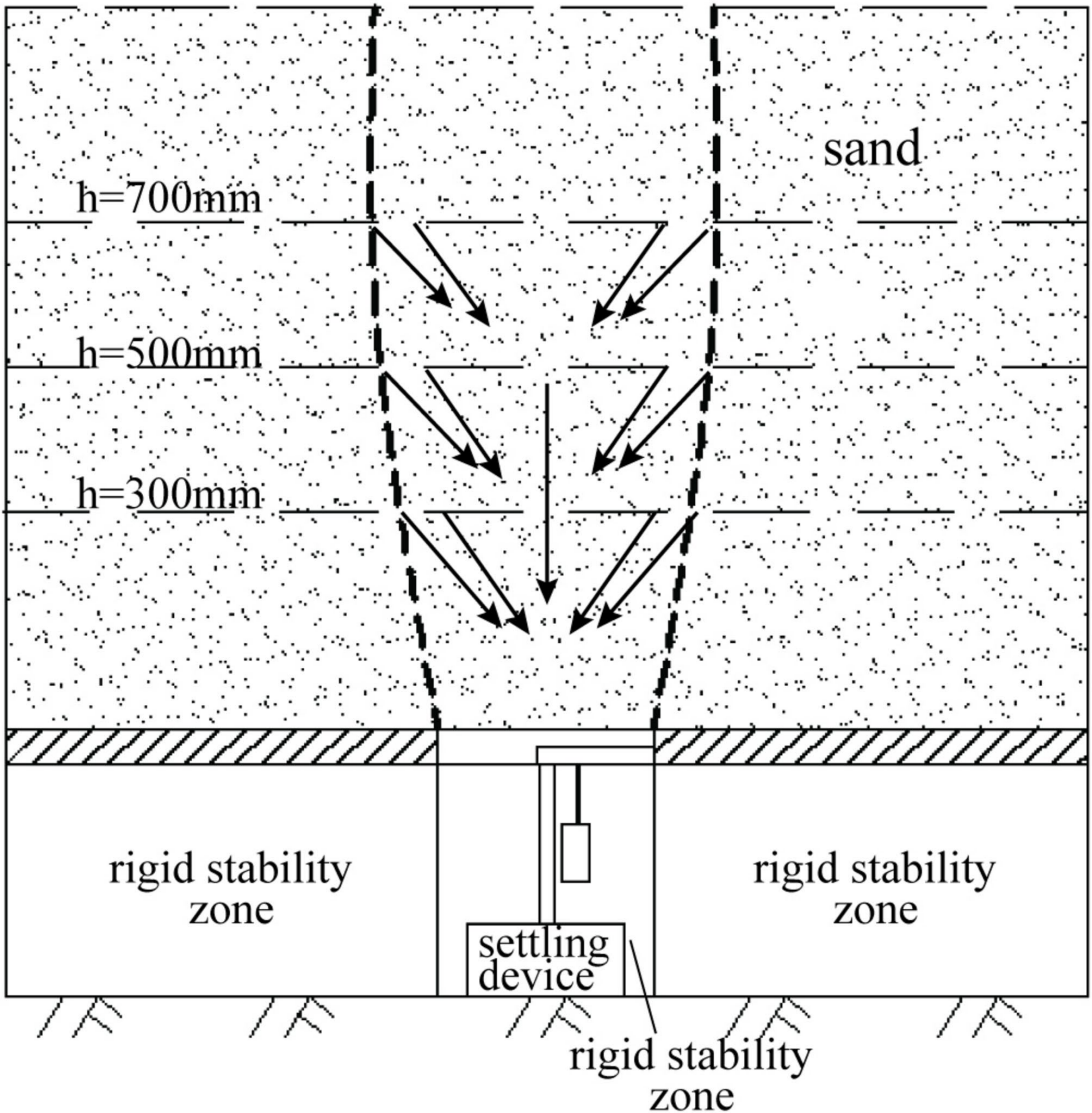
The figure of model tests.

Figure 9 describes the vertical displacement in the stable zone based on the displacement data from the sensors. As collapse progresses, sensors at a layout height of 300 mm (A1, A2, C1, and C2) in both tests A and C display the greatest vertical displacements. In contrast, sensors at a layout height of 500 mm (A3, A4, C3, and C4) present smaller vertical displacements. The least vertical displacement is observed at a layout height of 700 mm with sensors A5, A6, C5, and C6, indicating that the influence of collapse on the stable zone gradually decreases with height. This displacement gradient aligns with the soil arching mechanism observed in trapdoor tests. The descent of the settlement plate simulates the loss of underlying support, triggering shear-induced deformation in the overlying soil. The schematic illustration of soil displacement in the model experiment is depicted in Fig. 10. Initially, force chains form between soil particles, redistributing stresses to the periphery of the Subsidence zone and creating a transient arch structure. This arching effect reduces vertical displacement in upper layers (h = 700 mm) by transferring partial loads to stable regions. However, as collapse progresses (h = 300 mm), stress concentrations at the arch apex exceed the shear strength of soil, leading to localized arch failure and increased vertical displacement near the collapse source. The stable zone soil partially fills the collapsed periphery, further attenuating displacements at higher elevations.

**Fig. 11:**
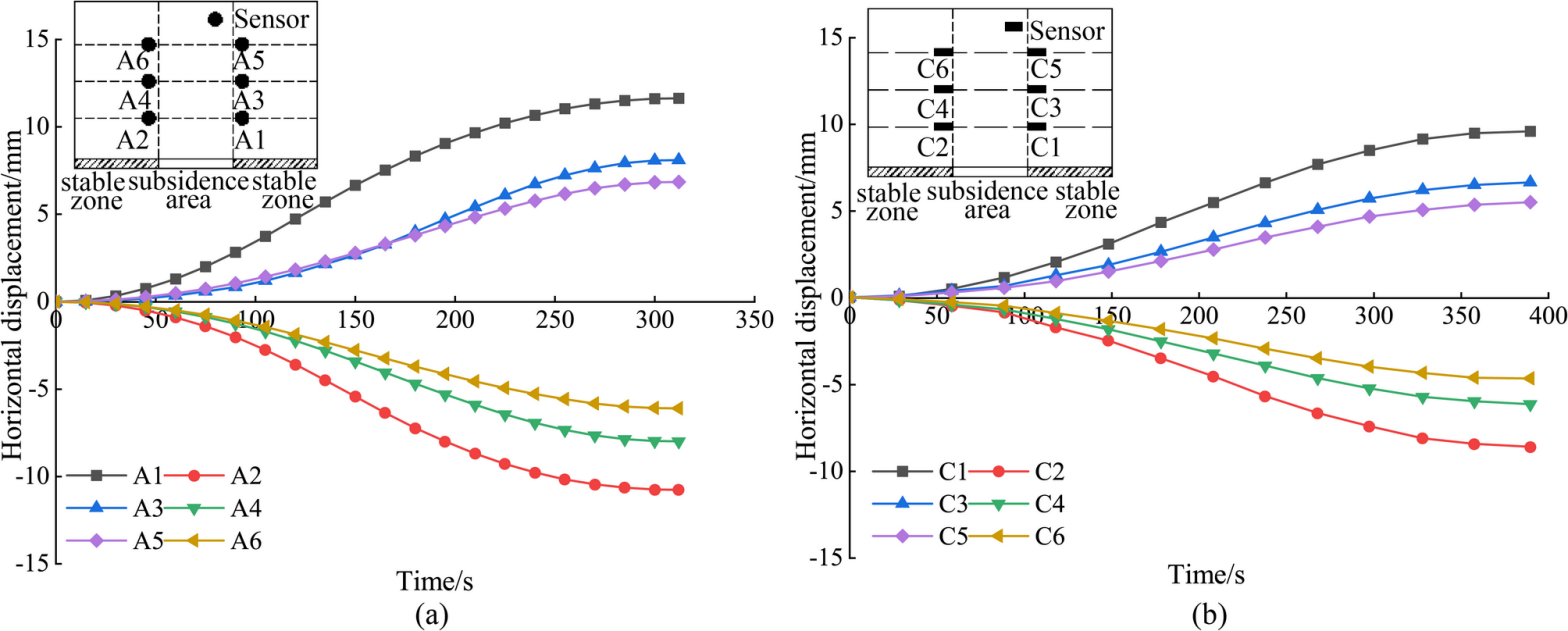
Analysis of horizontal displacement in the stable zone. (a) The horizontal displacement of the MEMS spherical monitoring devices in the stable zone; (b) the horizontal displacement of the MEMS sensors in the stable zone.

Figure 11 provides an analysis of horizontal displacements in the stable zone. For sensors located at a layout height of h = 300 mm (A1, A2, C1, and C2), the respective values are 10.77 mm, − 11.63 mm, 9.63 mm, and − 8.60 mm. At a layout height of h = 500 mm, the displacements for A3, A4, C3, and C4 are respectively 8.01 mm, − 8.10 mm, 6.58 mm, and − 6.14 mm. Moving to the height of h = 700 mm, the values for A5, A6, C5, and C6 are 6.11 mm, − 6.85 mm, 5.45 mm, and − 4.73 mm, respectively. These observations reveal a trend: the horizontal displacements for both the A and C test groups gradually decrease with an increase in the layout height, exhibiting a symmetrical pattern around the collapse source. Notably, the horizontal displacements between pairs A1 and A2, A5 and A6, and A9 and A10 progressively reverse direction during the collapse. A similar pattern is observed within the test C.

As illustrated in Fig. 11a, due to the influence of soil arching effect, the horizontal displacement is affected by the same effect as the vertical displacement in Fig. 9. This elucidates the significantly larger displacements recorded at sensors A1 and A2 compared to A5 and A6.

### Displacement analysis of subsidence zone

**Fig. 12:**
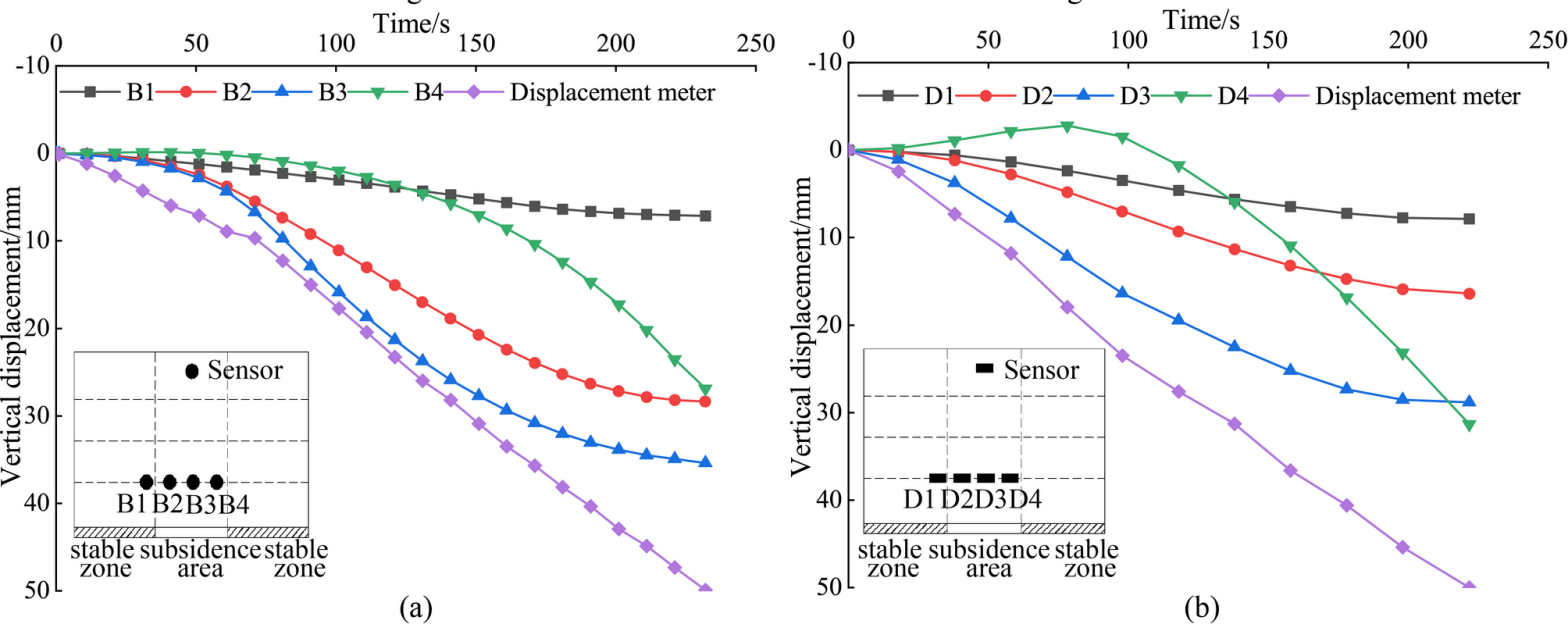
Analysis of vertical displacement in the subsidence zone (h = 300 mm). (a) The vertical displacement of the MEMS spherical monitoring devices in the subsidence zone; (b) the vertical displacement of the MEMS sensors in the subsidence zone.

**Fig. 13:**
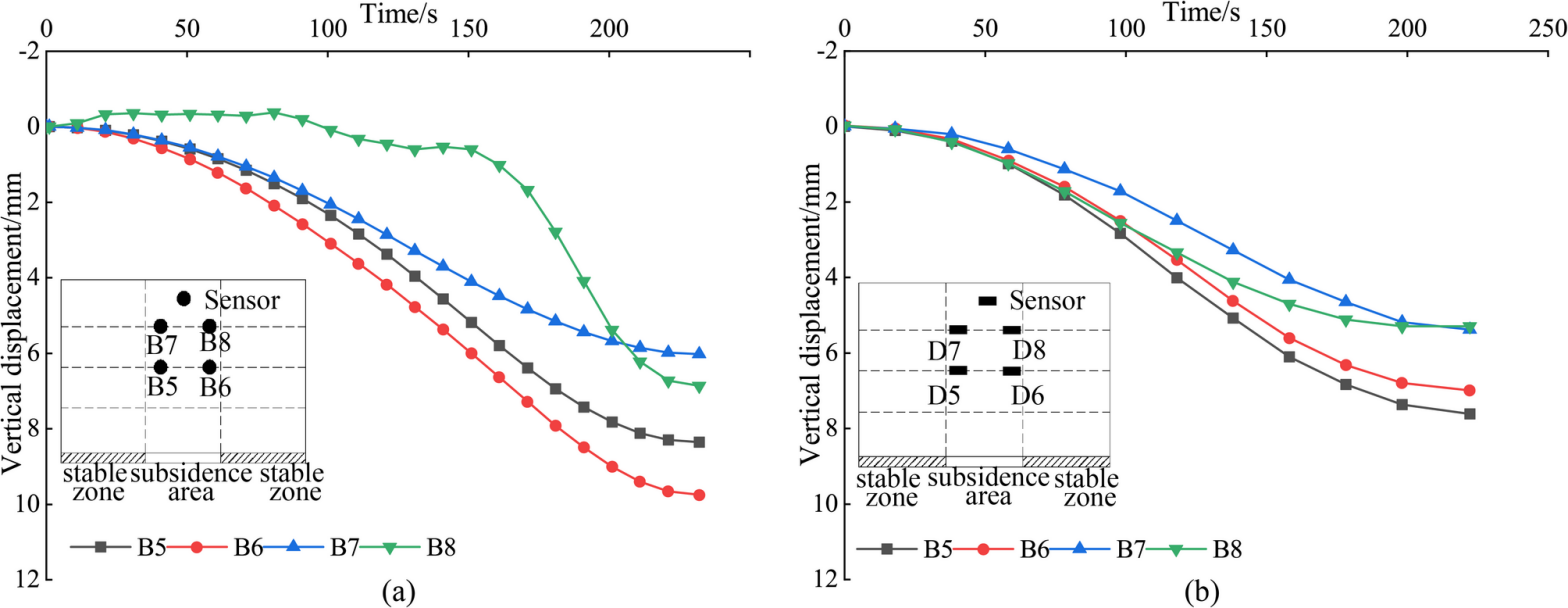
Analysis of vertical displacement in the subsidence zone (h = 500 and 700). (a) The vertical displacement of the MEMS spherical monitoring devices in the subsidence zone; (b) the vertical displacement of the MEMS sensors in the subsidence zone.

Vertical displacement analysis was conducted on sensors of the same type in the subsidence zone, considering different layout heights. The layout height of 300 mm corresponds to Fig. 12, while the heights of h = 500 mm and h = 700 mm correspond to Fig. 13. The Table 2 presents the final displacement values of the MEMS and MEMS spherical monitoring device at different height levels. The vertical displacement of sensors in the subsidence zone at the layout heights of h = 300 mm was significantly greater than that at h = 500 mm and h = 700 mm, indicating an increase in soil vertical displacement as it approaches the bottom of the subsidence zone. This occurs as a portion of the stable zone soil fills into the subsidence zone, resulting in a diminishing trend in the vertical displacement of soil in the upper subsidence zone.

For sensors of the same type at the same layout height, taking h = 300 mm as an example, the displacement of sensor B4 was similar to that of B2, but the displacement of D4 was significantly greater than that of the symmetrically positioned D2 sensor. This suggests that the soil cavities formed by tests B and D had different shapes, with the cavity formed by the D test group in the subsidence zone being irregular.

**Table 2:** The comparison of final displacement values between MEMS and MEMS spherical monitoring device.

Height	MEMS spherical monitoring device final displacement values/mm		MEMS sensor final displacement values/mm	
h = 300 mm	B1	7.1	D1	7.8
	B2	28.4	D2	16.4
	B3	35.5	D3	28.7
	B4	26.9	D4	31.3
h = 500 mm	B5	8.4	D5	7.6
	B6	9.8	D6	7.0
h = 700 mm	B7	6.0	D7	5.4
	B8	6.9	D8	5.3

**Fig. 14:**
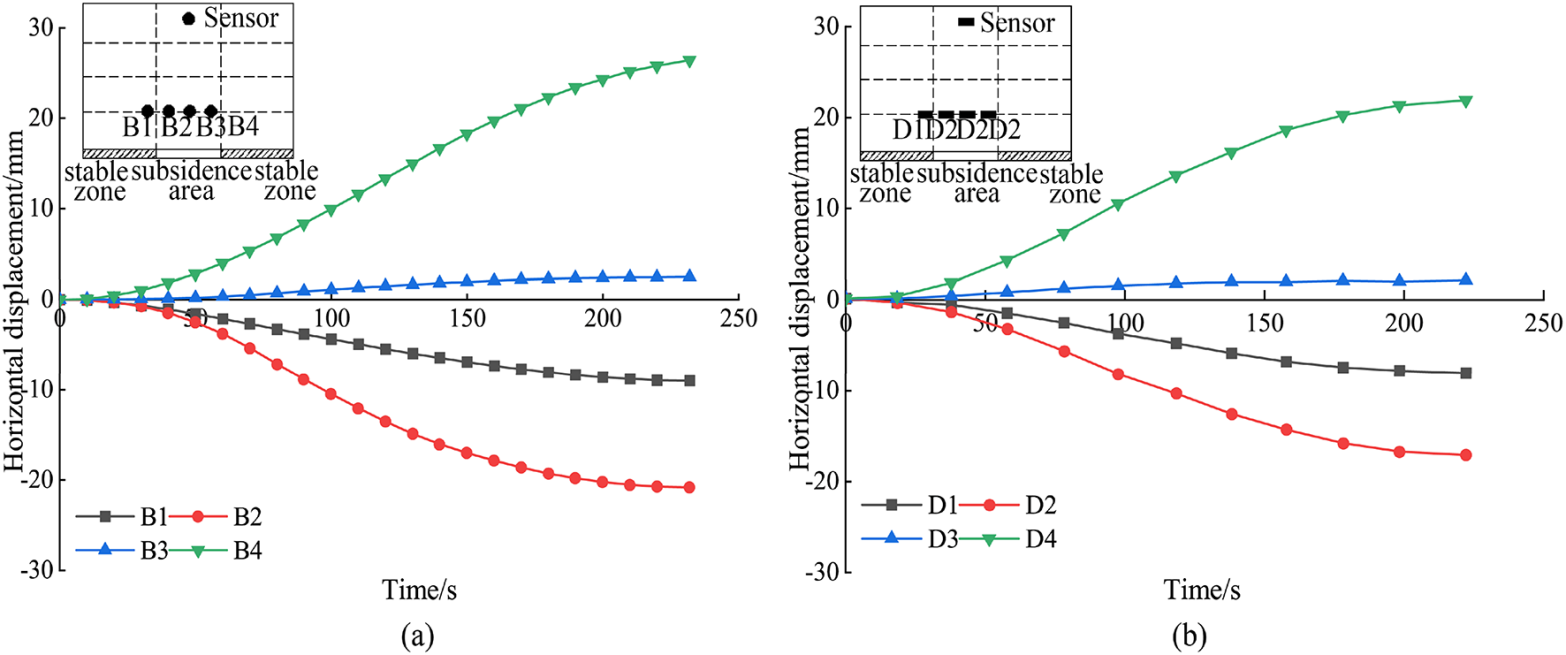
Analysis of horizontal displacement in the subsidence zone. (a) The horizontal displacement of the MEMS spherical monitoring devices in the subsidence zone; (b) the horizontal displacement of the MEMS sensors in the subsidence zone.

Figure 14 depicts the horizontal displacement curves for sensors placed at a height of h = 300 mm during tests B and D. A comparison of these curves reveals that sensors B3 and D3, which are positioned similarly, exhibit the smallest horizontal displacements. This trend likely results from the dominant influence of gravity, which causes a more pronounced downward movement of the soil at these locations. Additionally, the horizontal displacements for sensors B2 and B4, symmetrically positioned around the collapse center, are relatively similar. But B2 and B4 display larger dis-placements than B1, suggesting that positions off-center but closer to the collapse source experience greater horizontal displacement variability. Upon considering both vertical and horizontal displacements, it becomes evident that areas proximate to the collapse source demonstrate larger displacement variations. Interestingly, at the center of the collapse source, the vertical displacement is pronounced, while the horizontal displacement approaches zero. This indicates that the filling of stable zone soil does not affect the horizontal displacement at the center of the subsidence zone.

### Error analysis

The PIV (Particle Image Velocimetry) system utilized in this study is composed of a high-speed industrial CCD camera and Micro Vec V3 image processing software. This system allows for the analysis of particle image motion between consecutive frames, enabling the acquisition of particle velocity distribution. In the observed area and, consequently, the determination of vertical displacement data of the soil. the PIV device used in the model tests is shown in Fig. 8.

For the tests conducted in the stable zone (Tests A and C), vertical displacement data were extracted from the PIV cloud images using the six sensor locations as reference points. These points corresponded to the intersection points of soil heights of 300 mm, 500 mm, and 700 mm, and soil widths of 550 mm and 950 mm (Fig. 7). The vertical displacement data obtained from the six points extracted in PIV were considered as the true vertical displacement data of the soil. Additionally, the acceleration data from the six sensors in the stable zone were converted to vertical displacement data for the six reference points using the time-domain integration algorithm. These converted data served as the monitored values for soil vertical displacement. A comparison was made between the monitored values at the six positions and the true values obtained from Particle Image Velocimetry (PIV) for data analysis (Table 3).

Through calculations, it was found that the mean relative error of the MEMS sensors in the stable zone was 8.79%. However, for the MEMS spherical monitoring devices, the mean relative error was only 6.76%. This data indicates a 23.09% reduction in the mean relative error of vertical displacement in the stable zone when the MEMS spherical monitoring device is used compared to the MEMS sensors.

**Table 3:** The relative error value of the vertical displacement of the sensors in test A and test C.

Sensor ID	Vertical displacement		Vertical displacement (PIV)		Relative error		Mean relative error	
	MEMS spherical monitoring device /mm	MEMS sensor /mm	MEMS spherical monitoring device /mm	MEMS sensor /mm	MEMS spherical monitoring device /%	MEMS sensor /%	MEMS spherical monitoring device /%	MEMS sensor /%
1	5.9	7.2	6.7	6.3	– 11.94	14.28	6.76	8.79
2	5.6	6.9	5.2	6.3	7.69	9.52		
3	5.0	5.2	5.3	5.7	– 5.66	– 8.77		
4	5.5	5.9	5.2	5.5	5.77	7.27		
5	4.4	4.4	4.6	4.7	– 4.35	– 6.38		
6	4.1	4.3	3.9	4.6	5.13	6.52		

**Fig. 15:**
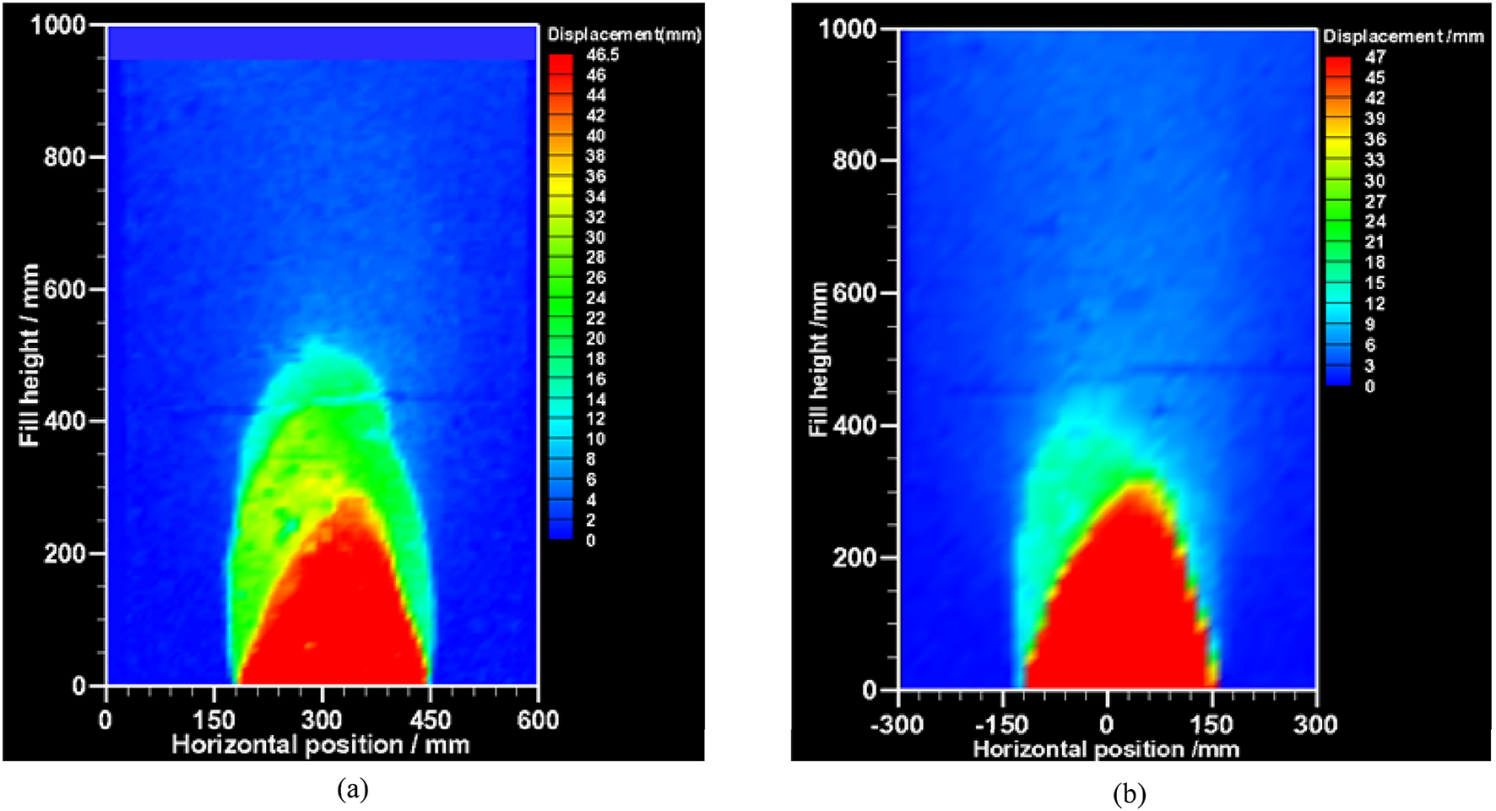
Vertical displacements in the subsidence zone of PIV (a) Vertical displacements in the subsidence zone of test B (PIV); (b) Vertical displacements subsidence zone of test D (PTV).

Figure 15 illustrates the displacement cloud images obtained from the PIV analysis for tests B and D, which were conducted in the subsidence zone. The color intensity within these images reflects the magnitude of the subsidence displacement, with darker colors indicating larger displacements. From the Figure, it is evident that the vertical displacement of the soil in Test B and Test D demonstrates a trend where the displacement becomes increasingly larger as it approaches the collapse source. Within the range of approximately 300 mm in soil height, the vertical displacement in both tests exhibits a relatively similar magnitude. However, as the soil height exceeds 300 mm, the vertical displacement in Test B significantly surpasses that in Test D.

To evaluate the vertical displacement in the subsidence zone, vertical displacement data were extracted from the PIV cloud images, using eight sensor locations illustrated in Fig. 6 as reference points. These points corresponded to heights of h = 300, 500, and 700 mm, and horizontal widths of L = 550, 650, 750, and 850 mm. The vertical displacement data extracted from these eight points in PIV were taken as the true vertical displacement data. Displacement obtained through the use of the time-domain integration algorithm from the eight sensors in the settlement zone served as the monitored values for the vertical displacement of subsurface soil. These monitored values were utilized as the reference data for vertical displacement in the subsequent error analysis of the subsidence zone (refer to Table 4).

Calculations reveal that the mean relative error of MEMS sensors in the subsidence zone was 10.70%, whereas the MEMS spherical monitoring devices displayed a mean relative error of 8.68%. The vertical displacement of the MEMS spherical monitoring devices in the subsidence zone showed an 18.87% reduction in the mean relative error compared to the MEMS sensors. This indicates the MEMS spherical monitoring devices potentially attributed to the ability to reduce the soil pressure impact on the monitoring circuit. The spherical design may also enable better soil integration, facilitating accurate soil displacement capture.

**Table 4:** The relative error value of the vertical displacement of the sensors in test B and test D.

Sensor ID	Vertical displacement		Vertical displacement (PIV)		Relative error		Mean relative error	
	MEMS spherical monitoring device /mm	MEMS sensor /mm	MEMS spherical monitoring device /mm	MEMS sensor /mm	MEMS spherical monitoring device /%	MEMS sensor /%	MEMS spherical monitoring device /%	MEMS sensor /%
1	7.1	7.8	6.7	7.1	5.97	9.86	8.68	10.70
2	28.3	16.3	25.5	17.5	10.98	– 6.86		
3	36.4	28.7	32.1	29.8	13.40	– 3.69		
4	26.9	31.2	24.8	37.9	8.47	– 17.68		
5	7.4	7.6	7.9	7.0	– 6.32	8.57		
6	9.7	6.9	10.4	6.1	– 6.73	13.11		
7	6.0	5.3	5.5	4.7	9.09	12.77		
8	6.4	5.2	5.9	4.6	8.47	13.04		

## Conclusions

This paper considers the factors affecting inertial sensors in a subsurface monitoring environment. A spherical MEMS monitoring device was developed and its applicability and advantages in karst collapse were validated through fixed-distance calibration tests and indoor model tests. Simultaneously, Particle Image Velocimetry (PIV) was employed to analyze the monitoring accuracy of the MEMS spherical monitoring devices and MEMS sensor in karst collapse model tests. The following conclusions were drawn.

1. The invention of a MEMS spherical monitoring device suitable for subsurface environments is validated through fixed-distance calibration tests, showing its ability to address the issue of sensors being unable to move synchronously with the subsurface soil due to soil pressure on the monitoring lines. Results indicate that compared to the MEMS sensor, the designed device reduces the average relative error in vertical displacement by 23.09% in stable zones and 18.87% in subsidence zones, demonstrating its ability to synchronize the moving soil in karst collapse monitoring.
2. The heterogeneous composition of karst soils, characterized by gravelly matrices and fragmented bedrock, introduces uncertainties in displacement measurements due to signal attenuation in MEMS sensors and kinematic constraints on device movement. To address these challenges, large-scale in-situ experiments in collapse-prone areas are imperative to validate the feasibility and robustness of MEMS spherical monitoring device under diverse geological conditions. Furthermore, integrating MEMS sensors with complementary monitoring technologies, such as fiber optic sensors and ground-penetrating radar, enables the development of multi-sensor fusion systems. These integrated frameworks demonstrate significant potential to enhance the accuracy, reliability, and resilience of slope stability monitoring and early warning systems.

## Data Availability

The datasets used in the present study are not publicly available, but they are available from the corresponding author upon reasonable request.

## References

[1] Gao, L., Shi, Y., Qiu, Y., Ma, C. & Zhou, A. The analyses of land use and prevention in High-Density main urban areas under the constraint of karst ground subsidence: study of Wuhan City, China. *ISPRS Int. J. Geo-Inf*. **12**, 425. 10.3390/ijgi12100425 (2023).

[2] Drobinina, E., Kovaleva, T. & Koriakina, A. The local variation of the overlying soils geotechnical properties in the karst susceptibility assessment. *Carbonates Evapor.* **35**, 79. 10.1007/s13146-020-00615-3 (2020).

[3] Lei, M., Zhou, W., Jiang, X., Dai, J. & Yan, M. Karst collapse monitoring. In *Atlas of Karst Collapses* 125–149 (Springer International Publishing, 2022). 10.1007/978-3-030-92912-1_8.

[4] Zhang, X. et al. Risk assessment and Spatial regulation on urban ground collapse based on geo-detector: a case study of Hangzhou urban area. *Nat. Hazards* **118**, 525–543. 10.1007/s11069-023-06016-8 (2023).

[5] Guan, Z., Jiang, X. & Gao, M. A calibration test of karst collapse monitoring device by optical time domain reflectometry (BOTDR) technique. In *Full Proceedings of the Thirteenth Multidisciplinary Conference on Sinkholes and the Engineering and Environmental Impacts of Karst, National Cave and Karst Research Institute, Carlsbad, New Mexico* 71–77 (2013). 10.5038/9780979542275.1115.

[6] Wu, H. et al. Optical Fiber-Based sensing, measuring, and implementation methods for slope deformation monitoring: a review. *IEEE Sens. J. PP* **2019**, 1–1. 10.1109/JSEN.2019.2891734 (2019).

[7] Liu, C. et al. Advances in automatic identification of road subsurface distress using ground penetrating radar: state of the Art and future trends. *Autom. Constr.* **158**, 586. 10.1016/j.autcon.2023.105185 (2024).

[8] Benedetto, A. & Pajewski, L. *Civil Engineering Applications of Ground Penetrating Radar* (Springer International Publishing, 2015). 10.1007/978-3-319-04813-0.

[9] Ho, M. B. Slope deformation monitoring in the Jiufenershan landslide using time domain reflectometry technology. *Landslides* (2019, accessed 5 Nov 2024). https://www.zhangqiaokeyan.com/journal-foreign-detail/0704023975279.html.

[10] Tao, T. et al. Design of a MEMS sensor array for dam subsidence monitoring based on dual- sensor cooperative measurements. *KSII Trans. Internet Inf. Syst.* **15**, 3554–3570. 10.3837/tiis.2021.10.005 (2021).

[11] Zhu, H. H. et al. Probing multi-physical process and deformation mechanism of a large- scale landslide using integrated dual-source monitoring. *Geosci. Front.* **15**, 101773. 10.1016/j.gsf.2023.101773 (2024).

[12] Wu, D. et al. Deformation monitoring of model foundation pit. *J. Appl. Sci. Eng.* **27**, 3349–3363. 10.6180/jase.202410_27(10).0015 (2024).

[13] Freddi, F., Mingazzi, L., Pozzi, E. & Aresi, N. Laboratory assessment of an In-Place inclinometer chain for structural and geotechnical monitoring. *Sensors* **23**, 8379. 10.3390/s23208379 (2023). 10.3390/s23208379 PMC10610819 37896473

[14] Jiao, C., Diao, Y., Han, J. & Zheng, G. Experimental research on a novel soil displacement monitoring system based on measurement unit cells (MUCs). *Measurement* **211**, 112605. 10.1016/j.measurement.2023.112605 (2023).

[15] Shentu, N. et al. Research on structure optimization and measurement method of a Large-Range deep displacement 3D measuring sensor. *Sensors* **20**, 1689. 10.3390/s20061689 (2020). 10.3390/s20061689 PMC7146746 32197396

[16] Kim, S. Y., Lee, J. S. & Hong, W. T. Subgrade assessment using automated dynamic cone penetrometer to manage Geo-infrastructures. *SMART Struct. Syst.* **27**, 861–870. 10.12989/sss.2021.27.5.861 (2021).

[17] Kim, S. Y. et al. Detection of roadbed layers in mountainous area using down-up-crosshole penetrometer and ground penetrating radar. *Measurement* **224**, 113889. 10.1016/j.measurement.2023.113889 (2024).

[18] Park, G., Lee, J. S., Kim, N., Lee, D. & Kim, S. Y. Effects of weight and drop height of hammer on dynamic cone penetration test in loose layer. *Measurement* **218**, 113198. 10.1016/j.measurement.2023.113198 (2023).

[19] Park, G., Lee, J. S., Kim, N. & Kim, S. Y. Hammer weight and dropping height effects on dynamic response in densely-packed geo-materials. *Transp. Geotech.* **44**, 101170. 10.1016/j.trgeo.2023.101170 (2024).

[20] Song, Y., Na, W., Jun, C. & Kim, S. Y. Comparative analysis of slope stability factors and a hydrological dataset for landslide assessment. *Natural Hazards*10.1007/s11069-024-06964-9 (2024).

[21] Park, G. et al. Instrumented dynamic cone penetrometer incorporated with time domain reflectometry. *Measurement* **206**, 112337. 10.1016/j.measurement.2022.112337 (2023).

[22] Kim, S. Y. et al. A review of UAV integration in forensic civil engineering: from sensor technologies to geotechnical, structural and water infrastructure applications. *Meas. J. Int. Meas. Confederation* **224**10.1016/j.measurement.2023.113886 (2024).

[23] Lee, J. S. et al. Geotechnical application of unmanned aerial vehicle (UAV) for Estimation of ground settlement after filling and compaction. *Transp. Geotechnics* **51**, 101517. 10.1016/j.trgeo.2025.101517 (2025).

[24] Nakata, Y. et al. DEM simulations on wave propagation around a subsurface cavity influenced by force chains. *J. Prod. Res.* **72**, 6 397–400. 10.11188/seisankenkyu.72.397 (2020).

[25] Ali, U., Otsubo, M., Ebizuka, H. & Kuwano, R. Particle-scale insight into soil arching under trapdoor condition. *Soils Found.* **60**, 1171–1188. 10.1016/j.sandf.2020.06.011 (2020).

[26] Otsubo, M., Ali, U. & Kuwano, R. Analyses on stability of subsurface cavity using discrete element method simulations. *J. Prod. Res.* **71**, 61051–61054. 10.11188/seisankenkyu.71.1051 (2019).

[27] Otsubo, M., Ali, U., Sato, T. & Kuwano, R. Development of arching in subsoil – trapdoor experiments and discrete element simulations. *SEISAN KENKYU*. **70**, 417–421. 10.11188/seisankenkyu.70.417 (2018).

[28] Britting, K. R. *Inertial Navigation System Analysis* 1th edn (Artech House, 1971).

[29] Guo, X., Sun, C., Wang, P. & Huang, L. A hybrid method for MEMS gyroscope signal error compensation. *Sens. Rev.* **38**, 517–525. 10.1108/SR-05-2017-0084 (2018).

[30] Hu, H., Zhang, L., Yan, H., Bai, Y. & Wang, P. Denoising and baseline drift removal method of MEMS hydrophone signal based on VMD and wavelet threshold processing. *IEEE Access.* **7**, 59913–59922. 10.1109/ACCESS.2019.2915612 (2019).

[31] Feng, K. et al. A new quaternion-based kalman filter for real-time attitude estimation using the two-step geometrically-intuitive correction algorithm. *Sensors* **17**, 2530. 10.3390/s17112530 (2017). 10.3390/s17092146 PMC5621018 28925979

[32] Guo, L., Song, J. & Ning, B. Positioning algorithm of mine used monorail crane locomotive based on strapdown inertial navigation. *Ind. Mine Autom.* **47**, 49–54. 10.13272/j.issn.1671-251x.2020080015 (2023).

[33] Kong, X., Yang, W., Luo, H. & Li, B. Application of stabilized numerical integration method in acceleration sensor data processing. *IEEE Sens. J.* **21**, 8194–8203. 10.1109/JSEN.2021.3051193 (2021).

[34] Wang, J. & Wang, Z. Lagrange polynomial fitting method for numerical integration of the vibration acceleration. *Noise Vib. Control*. **35**, 191–196 (2015).

[35] Zhang, R., Liu, X. & Qu, Z. The research of distance measurement method based on mobile phone MEMS accelerometer. In *2016 8th International Conference On Intelligent Human-Machine Systems And Cybernetics (IHMSC), vol. 2* 374–378 (IEEE, 2016). 10.1109/IHMSC.2016.131.

[36] Zhang, J., Xiu, C., Yang, W. & Yang, D. Adaptive threshold zero-velocity update algorithm under multi-movement patterns. *J. Beijing Univ. Aeronaut. Astronaut.* **44**, 636–644. 10.13700/j.bh.1001-5965.2017.0148 (2018).

[37] Wu, D., Wang, J. C. & Yang, Y. Application of MEMS sensor in physical test monitoring of karst collapse. *J. Saf. Environ.* **23**, 800–811. 10.13637/j.issn.1009-6094.2021.2002 (2023).

